# 

**Published:** 1969-01

**Authors:** 


### BRISTOL MEDICO-CHIRURGICAL JOURNAL

*(Incorporating the Medical Journal of the South West)*

RECEIVED
MAR 4 1970
National Library
of Medicine

Editors

N. J. Brown M.B., F.R.C.P.
A. B. Raper M.D., F.R.C.P.

1969
Vol. 84

Published quarterly under the auspices of the Bristol Medico-Chirurgical Society.

Printed by
F. Bailey & Son Ltd., Dursley, Glos.

CONTENTS

RICHARD SMITH JUNIOR AND HIS TIMES *A. L. Eyre-Brook* 1
THE MANAGEMENT OF SPINA BIFIDA IN BRISTOL .................. *J. B. M. Roberts* 12
PAROXYSMAL TACHYCARDIA, EPILEPSY, FRAGILITAS OSSIUM AND MENTAL RETARDATION ......... *J. Jancar* 17
THE SEVERAL AGES OF MEDICAL MAN ...... *Alan B. Raper* 19
NOTES AND NEWS ........................................................ 23
BOOKS RECENTLY ADDED TO THE LIBRARY .............. 24
THE CARE OF THE ELDERLY *W. H. Lloyd, A. W. Macara, P. S. Sinclair* 33
COLDHARBOUR FARM—THE FIRST FIVE YEARS ......*W. A. Heaton-Ward* 46
HOST RESISTANCE AND HOSPITAL INFECTION *W. A. Gillespie* 51
THE CHANGING MEDICAL CURRICULUM .................... 56
ANAPHYLACTOID PURPURA .............. *M. E. R. Stoneman* 61
MYELOID LEUKAEMOID REACTION IN A SURGICAL CASE ... *A. M. Ashraf* 72
SIXTY YEARS OF STOKE PARK HOSPITAL ......... *J. Jancar* 77
SOUTH WEST ORTHOPAEDIC CLUB ............................. 97
OBITUARY—J. A. POCOCK ............................................ 100
RECENTLY APPOINTED CONSULTANTS ....................... 23, 103
CHRONIC RHEUMATIC ENDOCARDITIS ......... *J. Goodwin* 107
SURGICAL ASPECTS OF RHEUMATIC HEART DISEASE *T. H. Sellors* 115
CARDIAC LESIONS IN CHILDREN WITH "HYPERCALCAEMIC" FACIES ................. *S. C. Jordan* 121
TWINS WITH RHEUMATIC DISEASES ......... *A. St. J. Dixon* 125
PHARMACOLOGICAL FIBRINOLYSIS ......... *G. R. Fearnley* 131
ARTHRITIS AND THE HEART .......................... *J. A. Cosh* 145
CLAUDICATION ........................................ *D. N. Walder* 153
LIPOPROTEIN DEFICIENCY DISORDERS ......... *J. K. Lloyd* 159
FINGER-PRINTING IN CONGENITAL HEART DISEASE ....................................... *T. J. David* 167
HOME CARE OF MYOCARDIAL INFARCTION *H. G. Mather* 171
THE CARE OF PEPTIC ULCER PATIENTS ...... *N. C. Tanner* 179
AORTIC VALVE REPLACEMENT *R. G. Charles and C. M. Morgans* 185
EVALUATION OF CLINICAL PHENOMENA IN PSYCHIATRY ........................................ *J. C. Little* 191
DRUGS AND LIVER DISEASE ......................... *A. E. Read* 199
PSEUDO-OBSTRUCTION OF THE SMALL BOWEL *F. Nahai* 209
ATRIAL BRADYCARDIA .............. *D. B. Shaw and D. Eraut* 213
SYMPOSIUM IN HONOUR OF CHARLES BRUCE PERRY ... 105-215

### BRISTOL MEDICO-CHIRURGICAL SOCIETY

*President* 1968-1969: Mr. A. L. Eyre-Brook

*President-elect*: Dr. F. J. W. Lewis

*Honorary Secretary*: Dr. A. T. M. Roberts, 8 Harley Place, Bristol 8.

*Honorary Treasurer*: Dr. J. Macrae, Ham Green Hospital, Pill.

The Society was founded in 1874. In 1883 publication of this Journal began, and has continued quarterly since then. During the years 1949 to 1962 it appeared under the title of "The Medical Journal of the South West".

The Society founded a medical library in 1890, which in 1892 was combined with that of the Bristol Medical School. This became the University of Bristol Medical Library in 1925, and an agreement in that year confirmed the privilege of Members to use the University Medical Library.

### THE BRISTOL MEDICO-CHIRURGICAL JOURNAL

*Editorial Committee*

EDITORS:

Dr. N. J. Brown, Southmead Hospital, Bristol.

Dr. A. B. Raper, Royal Infirmary, Bristol

MEMBERS:

Mr. H. B. Griffith

Dr. M. B. Lennard

Dr. A. E. Read

Mr. J. B. M. Roberts

Professor R. C. Wofinden

Dr. D. W. Wright

The Hon. Treasurer and Hon. Secretary of the Society.

SECRETARY:

Mr. A. E. S. Roberts, The Library, Medical School, University Walk, Bristol 8.

THE

BRISTOL MEDICO-CHIRURGICAL JOURNAL

*(Incorporating the Medical Journal of the South-West)*

Established 1883

Vol. 84 (i) January, 1968 No. 309

### *Contents*

| | | Page |
|---|---|---|
| RICHARD SMITH JUNIOR AND HIS TIMES | *A. L. Eyre-Brook* | 1 |
| THE MANAGEMENT OF SPINA BIFIDA IN BRISTOL | *J. B. M. Roberts* | 12 |
| PAROXYSMAL TACHYCARDIA, EPILEPSY, FRAGILITAS OSSIUM AND MENTAL RETARDATION .......... | *J. Jancar* | 17 |
| THE SEVERAL AGES OF MEDICAL MAN | *Alan B. Raper* | 19 |
| NOTES AND NEWS ........................................................ | | 23 |
| BOOKS RECENTLY ADDED TO THE LIBRARY .............. | | 24 |

*PUBLISHED QUARTERLY*

***ANNUAL SUBSCRIPTION 16/- POST FREE***

### BRISTOL MEDICO-CHIRURGICAL SOCIETY

President 1968-1969 : Mr. A. L. Eyre-Brook

President-elect : Dr. F. J. W. Lewis

Honorary Secretary : Dr. A. T. M. Roberts, 8 Harley Place, Bristol 8.

Honorary Treasurer : Dr. J. Macrae, Ham Green Hospital, Pill.

The Society was founded in 1874. In 1883 publication of this Journal began and has continued quarterly since then. During the years 1949 to 1962 i[illegible] appeared under the title of " The Medical Journal of the South West ".

The Society founded a medical library in 1890, which in 1892 was combine[illegible] with that of the Bristol Medical School. This became the University of Brist[illegible] Medical Library in 1925, and an agreement in that year confirmed th[illegible] privilege of Members to use the University Medical Library.

### THE BRISTOL MEDICO-CHIRURGICAL JOURNAL

*Editorial Committee*

*Editors :*

Dr. N. J. Brown, Southmead Hospital, Bristol
Dr. A. B. Raper, Royal Infirmary, Bristol

*Members :*

Mr. H. B. Griffith
Dr. M. B. Lennard
Dr. A. E. Read
Mr. J. B. M. Roberts
Professor R. C. Wofinden
Dr. D. W. Wright

*Secretary :*

Mr. A. E. S. Roberts, The Library, Medical School, University Wal[illegible] Bristol, 8.

The Hon. Treasurer and Hon. Secretary of the Society.

### NOTICE TO CONTRIBUTORS

The Journal is especially concerned to provide a place for the recording [illegible] medical thought, interests, and practice in and around Bristol. It therefo[illegible] welcomes articles that, as well as being of immediate interest to members, w[illegible] document the local and contemporary medical scene for our successors.

Original articles are invited provided that they have not been publishe[illegible] elsewhere. References in the text should be by author's name, with year [illegible] publication in parenthesis; they should be listed at the end in alphabetic[illegible] order. Illustrations should be supplied ready for reproduction. Line drawin[illegible] should be in indian ink on firm white paper or board. The author will [illegible] informed if it is found necessary to ask him to pay for the making of block[illegible] The Editorial Committee reserves the right to make literary corrections.

The following contributions will be especially welcome: notes of fort[illegible] coming events, meetings held, degrees conferred, staff appointments, honou[illegible] and public appointments and other local news.

### NOTES AND NEWS

### Pathologists in the South-West

The Wessex branch of the Association of Clinical Pathologists met at Musgrove Park Hospital, Taunton, on 9th November, 1968. The subjects discussed included tests in the control of anticoagulant therapy (Dr. R. D. Eastham), intermittent porphyria in a large Devonshire kindred (Dr. T. Hargreaves), the rare and as yet inexplicable acute fatty liver of pregnancy (Dr. E. E. Wood), and cytomegalovirus disease, both naturally occurring and following massive transfusion (Dr. I. D. Fraser and colleagues).

A new laboratory at the hospital, built at a cost of about £3,000, was officially opened by Dr. G. Stewart Smith.

### Obituary

Dr. R. M. Norman, Mr. J. A. Pocock.

### Recently Appointed Consultants

Dr. J. A. CLEMENT, M.B., B.S.(London), F.F.A.R.C.S., D.A., trained at Guy's Hospital qualifying in 1957. He subsequently worked there as a junior Anaesthetist and later at Leicester Royal Infirmary. In 1963 he came to Bristol to complete his anaesthetic training. His professional interests include the application of computers to Anaesthesia and Medicine; the sterilization of respiratory equipment; and problems associated with temperature changes during Anaesthesia. Apart from an involuntary interest in gardening he would like to indulge his interests in fishing and Rugby. He will continue to live in Easton in Gordano for the time being. He is married and has four children. His appointment will be maximum part-time.

Mr. CLOUGH, who is 35, was educated at Denstone College and at St. Mary's, graduating M.B., B.S. in 1957. After graduating he held house posts at St. Mary's and the Central Middlesex. He worked for three years in general surgery taking the F.R.C.S. in 1962. He then worked for two years in the orthopaedic department at St. Mary's Hospital, London before coming to Bristol, where for the past four years he has worked in the Bristol United Hospitals and at Winford Orthopaedic Hospital. His medical interests include the treatment of tibial fractures, the effects of compression on fracture healing, and the treatment of juvenile hallux valgus. His new appointment will be maximum part-time, divided between Frenchay and Winford hospitals.

Mr. RONALD W. HILES, M.B., Ch.B. (Bristol), F.R.C.S. (Ed), F.R.C.S. (Eng), has been appointed as Plastic Surgeon at Frenchay Hospital, where he was formerly senior registrar in the Department of Plastic and Jaw surgery. He qualified at Bristol in 1957 and worked in the R.A.F. Plastic Surgery Unit at Halton for several years. He is particularly interested in the treatment of injuries and diseases affecting the hand. He has four children and will live in the Stoke Bishop area. His appointment is maximum part-time.

### BOOKS RECENTLY ADDED TO THE LIBRARY

Grundrey, E. Dangerous people. The Spectator, 1967. Presented by Izal Ltd.
Hutchison's Clinical methods. Bailliere, 1968.
Crowley, M. F. A new look at nutrition. Pitman, 1967.
Gaddum's Pharmacology. O.U.P., 1968.
Foulk, W. T. Diseases of the liver. McGraw-Hill, 1968.
Starling's Principles of human physiology. Churchill, 1968.
The Medical Annual 1968. Presented by John Wright & Sons.
Chusid, J. G. & McDonald, J. J. Correlative neuroanatomy. Lange, 1968.
Fourman, L. P. R. & Royer, P. Calcium metabolism and bone. Blackwel[illegible] 1968.
Vogel, H. J. and others. Organizational biosynthesis. Academic Press, 1967.
Davies, O. L. Statistical methods in research. Oliver & Boyd, 1967.
Stewart, F. S. Bacteriology and immunity. Bailliere, 1968.
Kelman, G. R. & Nunn, J. F. Computer tables for blood oxygen tension an[illegible] content. Butterworth, 1968.
Ramachandran, G. N. ed. Treatise on collagen. Vols. 2A & 2B. Academ[illegible] Press, 1968.
Freeman, H. L. ed. Progress in behaviour therapy. 1968 Presented by Joh[illegible] Wright & Sons.
Read, A. E. A. ed. Biopsy procedures in clinical medicine. 1968. Presented b[illegible] John Wright & Sons.
Salter, R. H. Common medical emergencies. 1968. Presented by John Wrig[illegible] & Sons.
Elmore, D. T. Peptides and proteins. C.U.P. 1968.
White, A. and others. Principles of biochemistry. McGraw-Hill, 1968.
Race, R. R. & Sanger, R. Blood groups in man. Blackwell, 1968.
Molecular biology of viruses. 18th symposium of the Society for Gener[illegible] Microbiology. C.U.P., 1968.
MacKeith, R. & Bax, M. Studies in infancy. Heinemann, 1968.
Bio-organic chemistry. Readings from Scientific American. Freeman, 1968.
Illingworth, R. S. The normal child. Churchill, 1968.
Rafelson, M. E. & Binkley, S. B. Basic biochemistry. Macmillan, 1968.
Encyclopaedia of Urology, Vol. 7, Part 1—Malformations. Springer, 1968.
Advances in teratology. Vol. 3. Academic Press, 1968.
Richards, J. W. Introduction to industrial sterilization. Academic Press, 196[illegible]
Mahler, H. R. & Cordes, E. H. Basic biological chemistry. Harper, 1968.
Yearbook of endocrinology, 1967-68. Yearbook Publishers, 1968.
Progress in allergy. Vol. 12. Karger, 1968.
Winton, F. R. & Bayliss, L. E. Human physiology. Churchill, 1968.
With, T. K. Bile pigments. Academic Press, 1968.
Perspectives in virology. Vol. 5. Academic Press, 1967.
Sobotta, R. H. J. & Figge, F. H. J. Atlas of human anatomy. Vol. 1. Hafn[illegible] 1968.
Bell, G. H. and others. Textbook of physiology and biochemistry. Livin[illegible] stone, 1968.
Margoulies, M. ed. Protein and polypeptide hormones. Part 2. Excerp[illegible] Medica, 1968. Presented by the University of Liège.
Longacre, J. J. Craniofacial anomalies. Lippincott, 1968. Presented by [illegible] Research Foundation for Facially Crippled.

Kenyon, F. E. Psychiatric emergencies and the law. 1968. Presented by John Wright & Sons.

Crawford, M. A. ed. Comparative nutrition of wild animals. Zoological Society Symposium, 21, Academic Press, 1968.

Merck's Index of chemicals and drugs. 1968.

Oakley, W. G. and others. Clinical diabetes. Blackwell, 1968.

Printed by
F. Bailey & Son, Dursley, Glos.

